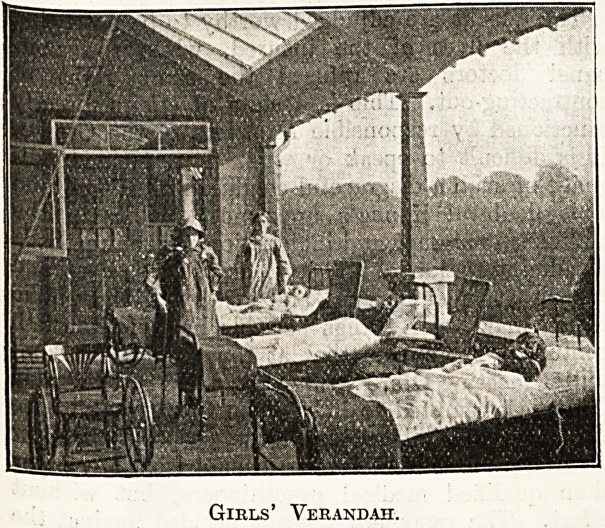# Opening of the Marple Cripples' Home

**Published:** 1913-11-15

**Authors:** 


### Opening of the Marple Cripples' Home.

The Crippled Children's Aid Society of Manchester and Salford has opened a new country nursing home or hospital at Marple, of which we publish photographs. The Earl of Derby performed the ceremony. Built at a cost of £5,000, the Chairman, Mr. T. Broadhurst, after thanking Lord Derby for his presence, appealed for £350 to clear off the debt and for support towards the yearly cost of maintenance, which he gave as £850.

Crippled Children's Nursing Home, Rose Hill, Marple.

Mr. C. B. Holmes, chairman of the institution, explained that the Home was not overlapping other institutions, but was doing work unprovided for elsewhere.

Lord Derbv, in his formal speech at the opening, said : There are two things in your work that strike me most. The first is that it is a work of personal service. The second is : You have recognised here, not only giving of parties to young crippled children—in itself a most

Girls' Verandah.

desirable object—but what I look upon as far more important than that, and that is the giving of children a lengthy time, under careful supervision; that makes it perhaps not only a temporary alleviation of their suffering, but may make it a permanent cure. I often think that in hospitals it is almost cruel the way that not only children, but people who are treated in hospitals, are brought up to a certain pitch of what they call cured, and yet are not given that extra time in the hospital or the home which may make all the difference as to whether it is really a permanent cure or not. It is not the fault of the hospitals or the doctors in those hospitals. The constant pressure for admission necessitates the freeing of beds. In this Home here you have recognised that there is a work to be done in giving longer treatment, and by extending, as you have, to a twelve months' sojourn in this new Home, you may in many cases have made what is only partial into a permanent recovery.

## Figures and Tables

**Figure f1:**
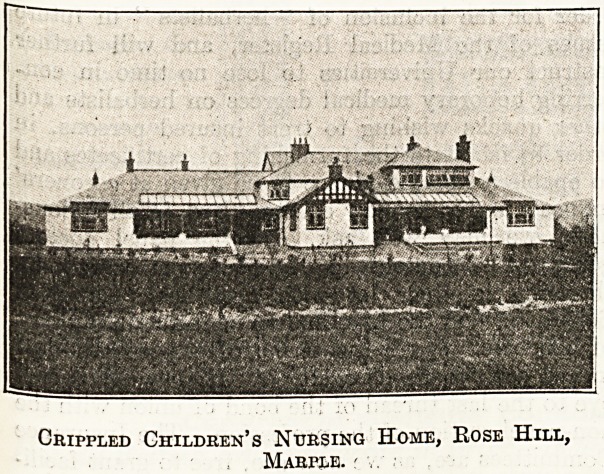


**Figure f2:**